# Prognostic utility of heart-type fatty acid-binding protein in patients with stable coronary artery disease and impaired glucose metabolism: a cohort study

**DOI:** 10.1186/s12933-020-0992-0

**Published:** 2020-02-10

**Authors:** Hui-Wen Zhang, Jing-Lu Jin, Ye-Xuan Cao, Hui-Hui Liu, Yan Zhang, Yuan-Lin Guo, Na-Qiong Wu, Ying Gao, Rui-Xia Xu, Qi Hua, Yan-Fang Li, Chuan-Jue Cui, Geng Liu, Qian Dong, Jing Sun, Jian-Jun Li

**Affiliations:** 1 State Key Laboratory of Cardiovascular Disease, Fu Wai Hospital, National Center for Cardiovascular Diseases, Chinese Academy of Medical Sciences and Peking Union Medical College, No 167 BeiLiShi Road, XiCheng District, Beijing, 100037 China; 2 Department of Cardiology, Xuanwu Hospital, Capital Medical University, Beijing, China; 3 Department of Cardiology, Beijing Anzhen Hospital, Capital Medical University, Beijing, China

**Keywords:** Heart-type fatty acid-binding protein, Impaired glucose metabolism, Coronary artery disease

## Abstract

**Background:**

Heart-type fatty acid-binding protein (H-FABP) is a novel marker of myocardial injury and has been reported to be associated with cardiovascular diseases (CVD) including patients with diabetes mellitus (DM). Unfortunately, its prognostic value in patients with CVD and impaired glucose metabolism (IGM) is unclear. The objective of this study was to investigate the prognostic value of H-FABP in CVD patients with IGM.

**Methods:**

A total of 4594 patients with angiography-proven coronary artery disease (CAD) were enrolled and divided into subgroup according to glucose metabolism status (normal glucose regulation [NGR], pre-DM, and DM). Baseline levels of H-FABP were measured using latex immunoturbidimetric method. The cardiovascular events (CVE) were defined as cardiovascular death, myocardial infarction, stroke and coronary revascularization. Cox regression and Kaplan–Meier analysis were used to evaluate the relations of H-FABP and glucose metabolism status to CVEs.

**Results:**

During the follow-up period with up to 7.1 years, 380 CVEs occurred. Patients with CVE had higher levels of H-FABP compared to those without CVE (p < 0.001). Interestingly, H-FABP levels were also elevated in DM and pre-DM groups compared with NGR group (p < 0.001), when combined glucose metabolism status with H-FABP stratification, patients in the highest tertile of H-FABP appeared to have higher risk of CVEs with pre-DM (adjusted hazard ratio [HR]: 1.855, 95% confidential intervals [CIs] 1.076–3.214; p = 0.033) and DM (adjusted HR: 2.560, 95% CIs 1.409–4.650; p = 0.002). The Kaplan–Meier curve indicated that DM patients with the highest H-FABP levels were associated with the greatest risk of CVEs (p < 0.05).

**Conclusions:**

Our data firstly showed that elevated H-FABP levels were associated with worse outcomes in CAD patients with pre-DM and DM, which provided the novel information that H-FABP might be a prognostic marker for clinical outcomes among patients with CAD and IGM.

## Background

It is well known that diabetes mellitus (DM) is a type of metabolic disease closely correlated with classic cardiovascular risk factors, such as obesity, hypertension, and dyslipidemia. Consequently, patients with DM are commonly complicated with higher risk of cardiovascular disease [1–3]. Pre-diabetes mellitus (pre-DM) is an intermediate status between normal glucose regulation (NGR) and DM, which is initially considered as a condition associated with the development of DM [4–6]. Recently, the attention for pre-DM has significantly been attracted due to its higher risk for coronary artery disease (CAD) and adverse cardiovascular events (CVE) [7–11]. Additionally, besides the atherosclerosis of large arteries can be easily induced by impaired glucose metabolism (IGM), previous studies have also provided the evidence supporting the relation of IGM to myocardial injury and silent ischemia among patients with IGM [12–14]. Furthermore, previous studies have also revealed that diabetic patients might suffer from cardiac dysfunction due to its complications such as diabetic cardiomyopathy or diabetic vasculopathy [15, 16].

Heart-type fatty acid binding protein (H-FABP), a novel cardiac marker, has been used as a diagnostic indicator of acute myocardial injury [17–19]. Previous studies have shown that H-FABP is not only a predictor of acute myocardial infarction (AMI) and adverse clinical events [19–22], but of other conditions as well. For example, series of studies have demonstrated that H-FABP elevated in patients with hypertension, dilated cardiomyopathy, heart failure, stroke, and pulmonary embolism [23–27]. Recently, plasma H-FABP levels were also found to be higher in diabetic patients, even in pre-diabetic status when compared with general population [13, 14, 28]. Akbal et al. [13] reported that serum H-FABP levels were significantly higher in diabetic patients with metabolic syndrome (MetS) than those without diabetic MetS. Besides, Basak et al. [28] found that H-FABP levels were increased in patients with IGM, and were positively correlated with carotid artery intima-media thickness (an intermediate phenotype for early atherosclerosis). Although the underlying mechanism of elevated H-FABP levels in relation to cardiac injury in patients with IGM is unclear, these data suggested that H-FABP might be a novel alternative index for detecting myocardial damage and early atherosclerosis, even a prognostic marker of future outcome in diabetic patients. The aim of the present study, therefore, was to investigate the role of H-FABP levels in predicting clinical outcomes in CAD patients with IGM.

## Methods

### Study population

**Fig. 1:**
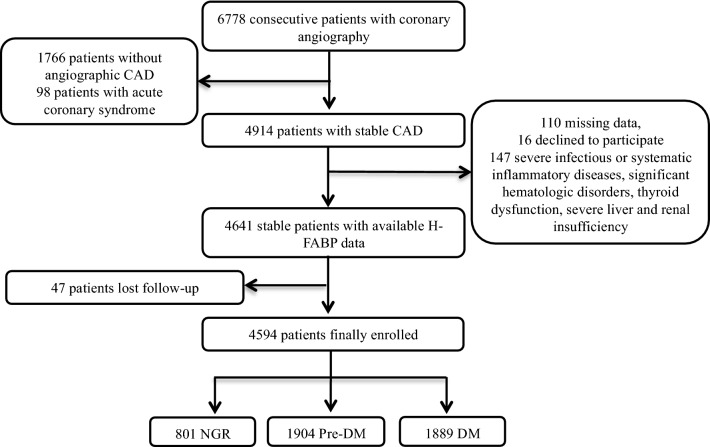
Study flowchart. *CAD* coronary artery disease, *H-FABP* heart-type fatty acid-binding protein, *NGR* normal glucose regulation, *Pre-DM* pre-diabetes mellitus, *DM* diabetes mellitus

From November 2011 to February 2017, 6778 consecutive patients underwent coronary angiography (CAG) because of angina-like chest pain and/or positive treadmill exercise test or clinically suspected CAD were enrolled from three medical centers. The study flowchart was shown as Fig. 1. Patients without H-FABP data or informed written consents, and patients with severe infectious or systematic inflammatory diseases, severe liver and renal insufficiency, significant hematologic disorders, and thyroid dysfunction were excluded. Finally, a total of 4594 patients were enrolled in analysis.

DM was diagnosed as fasting plasma glucose ≥ 7.0 mmol/L, the 2-h plasma glucose of the oral glucose tolerance test ≥ 11.1 mmol/L, or current therapy of hypoglycemic drugs or insulin. Pre-DM was defined as participants without self-reported DM or hypoglycemic treatment but with fasting plasma glucose ranging from 5.6 to 6.9 mmol/L, 2-h glucose ranging from 7.8 to 11.0 mmol/L, or hemoglobin A1c (HbA1c) level ranging from 5.7 to 6.4%. Then patients without DM or pre-DM were diagnosed as NGR [4]. Hypertension was defined as repeated systolic blood pressure ≥ 140 mmHg or diastolic blood pressure ≥ 90 mmHg (≥ three times), or self-reported hypertension, current antihypertensive treatments. Information of other disease history was collected from self-reported medical history. The body mass index (BMI) was calculated as weight (kg) divided by height (m) squared. The kidney function was measured as estimated glomerular filtration rate (eGFR) using Chronic Kidney Disease Epidemiology Collaboration (CKD-EPI) equation: 141 × min(SCr/κ, 1)^α^ × max(SCr/κ, 1)^−1.209^ × 0.993^Age^ × 1.018 [if female] × 1.159 [if Black] [29]. CAD was defined as the presence of coronary stenosis ≥ 50% at least one major artery segment assessed by two experienced physicians according to the results of CAG as mentioned in our previous studies [10]. The severity of coronary stenosis was assessed as Gensini Score (GS) system, which was defined as 1-point for < 25% stenosis, 2-point for 26–50% stenosis, 4-point for 51–75% stenosis, 8-point for 76–90% stenosis and 32-point for total occlusion. Then the score was multiplied by a factor that represents the importance of the lesion’s position in the coronary arterial system.

The CVEs included cardiovascular death, non-fatal myocardial infarction (MI), stroke, and coronary revascularization (CRV). Non-fatal MI was diagnosed as typical chest pain with positive cardiac troponins or typical changes of electrocardiogram serial. Stroke was diagnosed by the presence of typical symptoms and cerebral imaging examination. CRV included coronary percutaneous coronary artery intervention and/or bypass grafting. The participants were followed up at 6-months intervals by methods of interview or telephone conducted by trained physicians or nurses who were blinded to the clinical data.

### Statistical analysis

Mean ± standard deviation (SD) or median (inter-quartile range) was expressed for continuous data, and the qualitative data was shown as frequencies or percentage (n, %). Chi square test was used to compare the categorical data. T-test was performed for the normally distributed data to determine differences among groups. As for non-normally distributed data, the Mann–Whitney *U* test was performed for comparison between two-groups and the Kruskal–Wallis test was used for comparison of multi-groups as appropriate. Then we used a Cox proportional hazards analysis to explore the group differences among different levels of H-FABP and status of glucose metabolism. Finally, the event-free survival rates of CVEs among the subgroups according to H-FABP levels diabetic status were conducted by the Kaplan–Meier method and then compared by the log-rank test. Our statistical analyses were performed using SPSS software version 23.0 (SPSS, Inc., Chicago, IL, USA). The differences were considered significant if p < 0.05 in two-sided tests.

### Laboratory analysis

The venous blood samples were taken from each patient after a 12 h overnight fasting to collect baseline laboratory data prior to coronary intervention. Lipid profiles were determined by automatic biochemistry analyzer (Hitachi 7150, Tokyo, Japan). Detailed, total cholesterol (TC) and triglyceride (TG) were analyzed by enzymatic methods. Low-density lipoprotein cholesterol (LDL-C) concentration was determined by selective solublilization method (low-density lipid cholesterol test kit; Kyowa Medex, Tokyo). High-density lipoprotein cholesterol (HDL-C) concentration was measured by a homogeneous method (Determiner L HDL; Kyowa Medex, Tokyo). The concentrations of glucose were measured by enzymatic hexokinase method. Hemoglobin A1c (HbA1c) was measured using the Tosoh Automated Glycohemoglobin Analyzer (HLC-723G8). Plasma H-FABP levels were measured by Latex Immunoturbidimetric Method. This turbidometric immunoassay was performed using the Hitachi 7180 chemistry analyzer (reference interval < 5 ng/mL).

## Results

### Baseline characteristics

The baseline characteristics were provided in Table 1. A total of 4594 patients with stable CAD were finally included in our study, the mean age of participants was 58.2 ± 9.9 years and 3266 (71.9%) patients were male. According to the levels of H-FABP, patients were divided into 3 subgroups as Tertile 1 (< 1.80 ng/mL, n = 1685), Tertile 2 (1.80–2.70 ng/mL, n = 1452), and Tertile 3 (> 2.70 ng/mL, n = 1457). Data suggested that patients with higher H-FABP levels were older compared to those with lower H-FABP levels, but there was no significant difference in gender distribution with different H-FABP levels. In addition, patients with higher H-FABP levels had more frequent hypertension, diabetes as well as higher glucose, HbA1c, creatinine and lower eGFR levels (p < 0.05, respectively) compared to the lower ones. Moreover, we found that patients with elevated H-FABP levels showed increased Gensini score and incidence of CVEs (5.9% vs 8.3% vs 10.9%, p < 0.001). Overall, 380 (8.3%) CVEs occurred including 77 cardiovascular deaths, 49 non-fatal MI, 92 stroke and 162 CRV as shown in Additional file 1: Table S1. Notably, serum H-FABP levels were significantly elevated in event patients compared with non-event ones (mean: 2.95 ng/mL vs 2.41 ng/mL, p < 0.001). Meanwhile, patients with events were older, hypertensive and more likely to be current smokes (p < 0.05, respectively). In persons with events, there were higher level of glucose and HbA1c when compared with those without events (p < 0.05, respectively). There was no significant difference in family history of CAD, dyslipidemia and baseline treatments in hospital between the two groups (all p > 0.05).

**Table 1:** Baseline characteristics of patients with different H-FABP levels

Variables	Total (N = 4594)	Tertile 1 (N = 1685)	Tertile 2 (N = 1452)	Tertile 3 (N = 1457)	p value for trend
Baseline characteristics					
Age (years)	58.2 ± 9.9	54.7 ± 9.1	58.4 ± 9.2	62.0 ± 9.7	*< 0.001*
Male (n, %)	3266 (71.9%)	1206 (71.6%)	1041 (71.7%)	1019 (69.9%)	0.499
BMI (kg/m^2^)	25.92 ± 3.14	25.82 ± 2.98	25.91 ± 3.08	26.06 ± 3.36	0.114
Hypertension (n, %)	3005 (65.4%)	1041 (61.8%)	917 (63.2%)	1047 (71.9%)	*< 0.001*
Dyslipidemia (n, %)	4147 (90.3%)	1520 (90.2%)	1323 (91.1%)	1304 (89.5%)	0.337
Diabetes (n, %)	1889 (41.1%)	618 (36.7%)	566 (39.0%)	705 (48.4%)	*< 0.001*
Current smokers (n, %)	1438 (31.3%)	548 (32.5%)	468 (32.2%)	422 (29.0%)	0.065
Peripheral vascular disease (n, %)	59 (1.3%)	16 (0.9%)	19 (1.3%)	24 (1.6%)	0.222
Cerebrovascular disease (n, %)	145 (3.2%)	44 (2.6%)	46 (3.2%)	55 (3.8%)	0.177
Family history of CAD (n, %)	643 (14.0%)	258 (15.3%)	198 (13.6%)	187 (12.8%)	0.122
Laboratory data					
Triglyceride (mmol/L)	1.80 ± 1.23	1.82 ± 1.32	1.80 ± 1.15	1.78 ± 1.22	0.581
TC (mmol/L)	4.14 ± 1.17	4.15 ± 1.20	4.13 ± 1.09	4.14 ± 1.20	0.929
LDL-C (mmol/L)	2.51 ± 0.99	2.53 ± 1.08	2.51 ± 0.93	2.48 ± 0.95	0.260
HDL-C (mmol/L)	1.05 ± 0.29	1.05 ± 0.29	1.06 ± 0.29	1.06 ± 0.29	0.506
Glucose (mmol/L)	6.20 ± 2.04	6.12 ± 2.00	6.16 ± 1.99	6.33 ± 2.13	*0.016*
HbA1c (%)	6.54 ± 1.24	6.42 ± 1.22	6.50 ± 1.17	6.72 ± 1.30	*< 0.001*
Creatinine (μmol/L)	77.97 ± 17.30	74.17 ± 13.98	76.23 ± 14.62	84.10 ± 21.16	*< 0.001*
eGFR (mL/min/1.73 m^2^)	93.74 ± 9.12	97.38 ± 8.07	94.09 ± 8.09	89.16 ± 9.24	*< 0.001*
Treatments in hospital					
Aspirin (n, %)	4462 (97.1%)	1637 (97.2%)	1410 (97.1%)	1415 (97.1%)	0.997
β-Blokers (n, %)	3417 (78.3%)	1335 (79.2%)	1133 (78.0%)	1129 (77.5%)	0.477
Lipid-lowering mediation (n, %)	4269 (92.9%)	1551 (92.0%)	1348 (92.8%)	1370 (94.0%)	0.096
ACEI or ARB (n, %)	2157 (47.0%)	714 (42.4%)	669 (46.1%)	774 (53.1%)	*< 0.001*
Gensini score	34.50 ± 32.07	32.42 ± 30.33	33.70 ± 30.44	37.72 ± 35.24	*< 0.001*
Events (n, %)	380 (8.3%)	100 (5.9%)	121 (8.3%)	159 (10.9%)	*< 0.001*

Values are expressed as the mean ± SD or n (%). Italic values indicate statistical significance

*CAD* coronary artery disease, *BMI* body mass index, *NGR* normal glucose regulation, *Pre-DM* pre-diabetes mellitus, *DM* diabetes mellitus, *TC* total cholesterol, *LDL-C* low-density lipoprotein cholesterol, *HDL-C* high-density lipoprotein cholesterol, *HbA1c* glycated hemoglobin A1c, *H-FABP* heart-fatty acid binding protein, *ACEI* angiotensin converting enzyme inhibitors, *ARB* angiotensin receptor blocker, *eGFR* estimated glomerular filtration rate

In Table 2, patients were divided into three subgroups as NGR (n = 801, 17.4%), pre-DM (n = 1904, 41.4%) and DM (n = 1889, 41.1%). Across all three conditions, patients with pre-DM and DM were older, had higher BMI, and higher rate of hypertension and dyslipidemia (all p < 0.05) but less to be male and current smokers as compared with controls. H-FABP levels were significantly higher in the subjects with pre-DM and DM compared with those in NGR group (p < 0.001). Baseline glucose and HbA1c were lower in the controls as compared with both groups of pre-DM and DM. Additionally, HDL-C cholesterol was lower and TG, TC levels were higher in subjects with IGM (all p < 0.05). The serum creatinine levels did not differ significantly in the three groups (p = 0.532) but eGFR were decreased in patients with IGM compared to ones in NGR group (p < 0.001). Importantly, diabetic patients had higher Gensini score compared with the controls (p < 0.001). In total cohort, not surprisingly, patients in DM group had more than 1.5 times increased incidence of CVE compared with NGR group (11.1% vs 5.6%, p < 0.001) but no difference in the occurrence of CVEs between NGR and pre-DM group (5.6% vs 6.6%, p > 0.05).

**Table 2:** Baseline characteristics according to status of glucose metabolism

Variables	Total (N = 4594)	NGR (N = 801)	Pre-DM (N = 1904)	DM (N = 1889)	p value for trend
Baseline characteristics					
Age (years)	58.2 ± 9.9	55.2 ± 9.9	58.3 ± 9.5	59.3 ± 9.7	< 0.001*
Male (n, %)	3266 (71.9%)	617 (77.0%)	1346 (70.7%)	1303 (69.0%)	< 0.001*
BMI (kg/m^2^)	25.92 ± 3.14	25.51 ± 3.01	25.69 ± 2.96	26.38 ± 3.42	*0.006*
Hypertension (n, %)	3005 (65.4%)	461 (57.6%)	1200 (63.0%)	1344 (71.1%)	*< 0.001*
Dyslipidemia (n, %)	4147 (90.3%)	701 (87.5%)	1745 (91.6%)	1701 (90.0%)	0.004*
Current smokers (n, %)	1438 (31.3%)	332 (41.4%)	704 (37.0%)	402 (21.3%)	*< 0.001*
Peripheral vascular disease (n, %)	59 (1.3%)	6 (0.7%)	18 (0.9%)	35 (1.9%)	*0.015*
Cerebrovascular disease (n, %)	145 (3.2%)	24 (3.0%)	60 (3.2%)	61 (3.2%)	0.951
Family history of CAD (n, %)	643 (14.0%)	137 (17.1%)	255 (13.4%)	251 (13.3%)	0.020*
Laboratory data					
Triglyceride (mmol/L)	1.80 ± 1.23	1.67 ± 1.14	1.73 ± 1.00	1.93 ± 1.46	*< 0.001*
TC (mmol/L)	4.14 ± 1.17	4.03 ± 1.03	4.19 ± 1.17	4.14 ± 1.22	< 0.001*
LDL-C (mmol/L)	2.51 ± 0.99	2.45 ± 1.01	2.54 ± 0.97	2.50 ± 0.95	*0.284*
HDL-C (mmol/L)	1.05 ± 0.29	1.07 ± 0.31	1.08 ± 0.30	1.03 ± 0.28	*0.001*
Glucose (mmol/L)	6.20 ± 2.04	4.79 ± 0.42	5.32 ± 0.65	7.69 ± 2.41	< 0.001*
HbA1c (%)	6.54 ± 1.24	5.37 ± 0.24	5.05 ± 0.26	7.64 ± 1.23	< 0.001*
H-FABP (ng/mL)	2.45 ± 1.86	2.10 ± 1.13	2.41 ± 1.71	2.64 ± 2.21	< 0.001*
Creatinine (μmol/L)	77.97 ± 17.30	78.55 ± 16.28	78.40 ± 18.94	77.29 ± 15.95	0.532
eGFR (mL/min/1.73 m^2^)	93.74 ± 9.12	95.77 ± 8.87	93.80 ± 8.64	92.81 ± 9.56	< 0.001*
Treatments in hospital					
Aspirin (n, %)	4462 (97.1%)	776 (96.9%)	1861 (97.7%)	1825 (96.6%)	0.103
β-Blokers (n, %)	3417 (78.3%)	595 (74.3%)	1466 (77.0%)	1536 (81.3%)	*< 0.001*
Lipid-lowering mediation (n, %)	4269 (92.9%)	731 (91.3%)	1786 (93.8%)	1752 (92.7%)	0.058
ACEI or ARB (n, %)	2157 (47.0%)	334 (41.7%)	877 (46.1%)	946 (50.1%)	*< 0.001*
Gensini score	34.50 ± 32.07	30.94 ± 28.02	32.16 ± 29.54	38.38 ± 35.56	*< 0.001*
Events (n, %)	380 (8.3%)	45 (5.6%)	125 (6.6%)	210 (11.1%)	*< 0.001*

Values are expressed as the mean ± SD or n (%). Italic values indicate statistical significance. *Indicate statistical significance between NGR and Pre-DM

*CAD* coronary artery disease, *BMI* body mass index, *TC* total cholesterol, *LDL-C* low-density lipoprotein cholesterol, *HDL-C* high-density lipoprotein cholesterol, *HbA1c* glycated hemoglobin A1c, *H-FABP* heart-fatty acid binding protein, *ACEI* angiotensin converting enzyme inhibitors, *ARB* angiotensin receptor blocker, *NGR* normal glucose regulation, *Pre-DM* pre-diabetes mellitus, *DM* diabetes mellitus, *eGFR* estimated glomerular filtration rate

### H-FABP, glucose metabolism and events

The relationship between H-FABP, glucose metabolism and CVEs was explored by stratifying the patients into subgroups according to the tertiles of H-FABP and glucose metabolism status. As shown in In Additional file 1: Table S2, data from the Cox proportional-hazards model indicated that the highest tertile of H-FABP levels were associated with increased risk of CVEs [unadjusted hazard ratios (HR): 1.670; 95% confidence intervals (CIs) 1.299–2.146, p < 0.001]. After adjustment of age, gender, hypertension, dyslipidemia, BMI, current smoking, family history of CAD, Gensini score and eGFR, the results retained (adjusted HR: 1.335; 95% CIs 1.011–1.762, p = 0.041). With regard to the glucose metabolism, only patients in DM group showed association with CVEs at HR with 1.608 (95% CIs 1.151–2.248, p = 0.005).

Next, we performed subgroups analysis to further explore the correlation between pre-DM, DM and H-FABP with CVEs. As listed in Table 3, in unadjusted model, both patients with pre-DM and DM in the highest tertile of H-FABP showed positive association with CVEs and these findings persisted in adjusted models in pre-DM patients (HR: 1.335, 95% CIs 1.059–1.772, p = 0.039) and DM patients (HR: 1.322, 95% CIs 1.035–1.730, p = 0.040). Additionally, we further investigate the relationship between IGM and H-FABP with CVEs by combining the status of glucose metabolism with H-FABP stratification. Hence, the patients were divided into 9 subgroups. In Table 4, the Cox regression analysis after adjustment for the confounders according to both glucose metabolism and H-FABP status indicated that patients with pre-DM plus the highest tertile of H-FABP levels showed 1.855-fold higher risk of CVE (95% CIs 1.076–3.214, p = 0.033) compared with the reference group (NGR plus Tertile 1 of H-FABP). What’s more, patients in DM group had significantly increased risk of CVEs compared with the reference group (NGR plus Tertile 1 of H-FABP) in all tertiles of H-FABP (Tertile 1: adjusted HR: 2.131, 95% CIs 1.157–3.925, p = 0.015; Tertile 2: adjusted HR: 2.171, 95% CIs 1.179–3.997, p = 0.013; Tertile 3: adjusted HR: 2.560, 95% CIs 1.409–4.650, p = 0.002).

**Table 3:** H-FABP levels in relation to cardiovascular events in patients with pre-DM and DM

IGM	HRs (95% CIs)			
	Unadjusted	p value	Adjusted^a^	p value
Pre-DM				
Tertile 1	Reference	–	Reference	–
Tertile 2	1.206 (0.762–1.908)	0.424	0.971 (0.556–1.234)	0.728
Tertile 3	1.864 (1.203–2.890)	*0.005*	1.335 (1.059–1.772)	*0.039*
DM				
Tertile 1	Reference	**–**	Reference	**–**
Tertile 2	1.142 (0.820–1.608)	0.628	1.012 (0.795–1.423)	*0.772*
Tertile 3	1.631 (1.161–2.362)	*0.021*	1.322 (1.035–1.730)	*0.040*

Italic values indicate statistical significance

*Pre-DM* pre-diabetes mellitus, *DM* diabetes mellitus, *H-FABP* heart-type fatty acid-binding protein, *HRs* hazard ratios, *CIs* confidential intervals

^a^Adjusted for age, gender, hypertension, dyslipidemia, body mass index, current smoking, family history of CAD and Gensini score, eGFR

**Table 4:** H-FABP levels in relation to cardiovascular events in patients with different glucose metabolism status

Glucose metabolism status	HRs (95% CIs)			
	Unadjusted	p value	Adjusted^a^	p value
NGR				
Tertile 1	Reference	–	Reference	–
Tertile 2	2.101 (0.998–4.215)	0.053	2.016 (0.994–4.088)	0.052
Tertile 3	1.563 (0.713–3.427)	0.265	1.347 (0.611–2.967)	0.460
Pre-DM				
Tertile 1	1.303 (0.696–2.477)	0.418	1.211 (0.635–2.439)	0.448
Tertile 2	1.498 (0.802–2.796)	0.205	1.395 (0.748–2.579)	0.312
Tertile 3	2.382 (1.295–4.382)	*0.005*	1.855 (1.076–3.214)	*0.033*
DM				
Tertile 1	2.590 (1.413–4.746)	*0.002*	2.131 (1.157–3.925)	*0.015*
Tertile 2	2.755 (1.511–5.023)	*0.001*	2.171 (1.179–3.997)	*0.013*
Tertile 3	3.529 (1.976–6.302)	*< 0.001*	2.560 (1.409–4.650)	*0.002*

Italic values indicate statistical significance

*NGR* normal glucose regulation, *Pre-DM* pre-diabetes mellitus, *DM* diabetes mellitus, *H-FABP* heart-type fatty acid-binding protein, *HRs* hazard ratios, *CIs* confidential intervals

^a^Adjusted for age, gender, hypertension, dyslipidemia, body mass index, current smoking, family history of CAD and Gensini score, eGFR

**Fig. 2:**
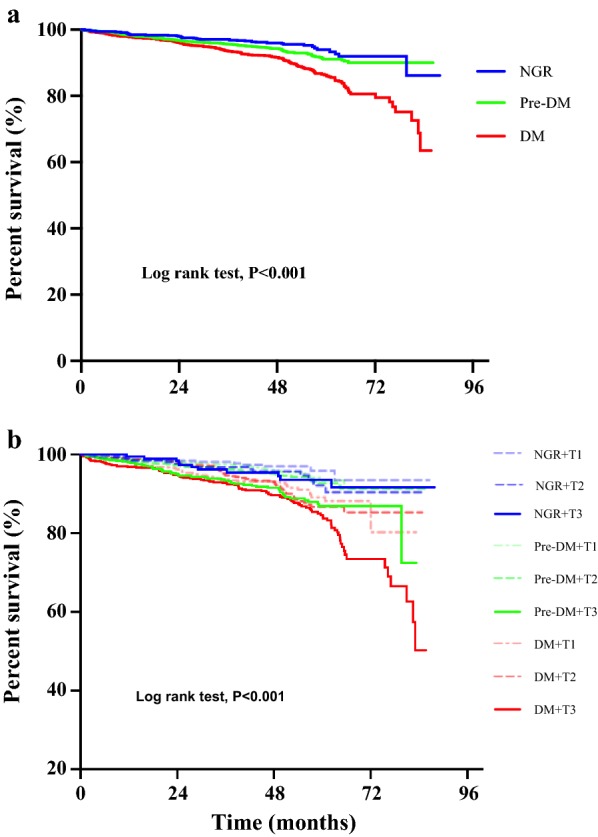
Kaplan–Meier survival analysis. **a** Showed the Kaplan–Meier survival curves of cardiovascular event in according to status of glucose metabolism; **b** showed the Kaplan–Meier survival curves in 9 subgroups according to the Heart-type fatty acid-binding protein (H-FABP) tertiles and status of glucose metabolism. *NGR* normal glucose regulation, *Pre-DM* pre-diabetes mellitus, *DM* diabetes mellitus

Finally, we performed the Kaplan–Meier curves for the subgroups according to the H-FABP tertiles and glucose metabolism status as shown in Fig. 2. Figure 2a demonstrated that patients with DM were associated with the higher risk of adverse events. Notably, the Kaplan–Meier curves demonstrated that patients with DM in the highest H-FABP group were associated with the greatest risk of CVEs (Fig. 2b).

## Discussion

As compared with what we known about H-FABP, a novel biomarker for myocardial injury in CAD, little is known about its prognostic value for patients with IGM. Data from the present study firstly indicated that H-FABP levels were increased in patients with IGM and importantly, H-FABP was independently associated with worse clinical outcomes in CAD patients with pre-DM and DM. This result provided novel information that elevated circulating levels of H-FABP might be a prognostic predictor of adverse events in CAD patients with mild abnormal glucose metabolism.

As is well known, DM is a worldwide disease results in poor clinical outcomes, and is associated with large health-care costs. Nowadays, pre-DM has been attracted more attention because of its close association with increased cardiovascular risks [7–11]. In our previous studies conducted by Liu et al. [9] and Jin et al. [10], patients with pre-DM were more likely to have higher CVE risk when combined with other risk such as hypertension. What’s more, the myocardial injury is reported to be another health issue in diabetic patients. Several studies have provided the evidence of myocardial damage in the early asymptomatic period among patients with IGM [12–14]. Additionally, some reports also indicated that diabetic patients could have heart damage in results of its complications like diabetic cardiomyopathy [15, 16]. Therefore, a further understanding of the cardiovascular risk associated with IGM will help to risk stratification and preventive strategies in pre-diabetic patients.

H-FABP is a small (15 kDa) cytoplasmic protein which transfers free fatty acids from the plasma membrane in cardiomyocytes. It can be rapidly released into circulation in cases of myocardial injury and is shown to be more sensitive than myoglobin and cardiac troponins [19, 22, 30]. Indeed, H-FABP is a newly identified biomarker for acute myocardial damage and an independent predictor of adverse cardiac events [19–22]. In addition, previous studies have also shown that H-FABP levels increased in patients with several vascular diseases, and also in MetS patients with IGM [13, 14, 28]. For instance, Akbal et al. [13] reported that serum H-FABP levels were significantly higher in diabetic patients with MetS than patients without diabetic MetS, indicating its promise as a marker for detection of cardiac injury during the early asymptomatic period in diabetic patients. Another study from provided that patients with insulin resistance and MetS tended to have higher H-FABP levels, suggested that the early stages of metabolic disorder might be exposed to myocardial damage and susceptible to silent heart failure [14]. In addition, Basak et al. [28] found H-FABP levels were increased in patients with impaired glucose metabolism, and serum H-FABP levels were positively correlated with carotid artery intima-media thickness, an intermediate phenotype for early atherosclerosis. These studies showed that serum H-FABP measurements might be used for determination of subclinical myocardial injury or subclinical atherosclerosis in diabetic patients. Therefore, we hypothesized that H-FABP could also be a useful marker for predicting the clinical outcomes in high-risk population of diabetic patients with CAD.

In the present study, we conducted a multicenter, prospective cohort with a follow-up period for up to 7.1 years to explore the prognostic role of H-FABP in CAD patients with IGM. Firstly, H-FABP levels appeared to be increased in patients with pre-DM and DM compared with normal group. There was also a significantly elevation of H-FABP levels in patients with CVEs in comparison to ones without CVEs. The hypothesized mechanism underlying the elevation of H-FABP in CAD patients with IGM has not been thoroughly studied. In a previous study of experimental diabetic model, it was reported that the H-FABP content of rat heart was marked increased, which might cause faster utilization of fatty acid in the diabetic heart [31]. Besides, diabetic patients could have structural and/or functional modifications in the myocardial tissue such as lacking of insulin sensitivity and glucose assimilation. As a compensatory mechanism, H-FABP may increase when fatty acid uptake increases in heart to supply more adenosine triphosphate (ATP) [15, 16]. On the other hand, there is evidence that abnormal of cardiac microvascular circulation caused by metabolic disorders might induce cardiomyocyte injury and impair cardiac function [32]. Other causes such as inflammation, oxidative stress, and apoptosis increased the permeability of cardiomyocyte, contributed to the release and serum elevation of H-FABP levels in patients with cardiac damage [33]. These plausible evidences may be the explanations why H-FABP elevated in CAD patients with IGM.

In literature, the development and progression of atherosclerotic disease is modulated by many cardio-metabolic risk factors. Interestingly, in this study, we found no difference of the CVE incidence between patients with NGR and ones with pre-DM (5.6% vs 6.6%, p > 0.05). When the glucose metabolism status was incorporated in H-FABP stratification, data revealed that the higher tertile of H-FABP patients were associated with higher risk of CVEs in pre-DM (adjusted HR: 1.855, 95% CIs 1.076–3.214, p = 0.033), suggesting that H-FABP could be an independently index of adverse outcomes in CAD patients with pre-DM. To sum up, we not only analyzed the prognosis of H-FABP, but also further demonstrated the impacts of elevated H-FABP levels plus different glucose metabolic status. As the main novel findings of our study, when status of glucose metabolism was incorporated in H-FABP stratification, patients in the highest level of H-FABP group were associated with higher risk of CVEs in both pre-DM group (adjusted hazard ratio [HR]: 1.855, 95% confidential intervals [CIs] 1.076–3.214; p = 0.033) and DM (adjusted HR: 2.560, 95% CIs 1.409–4.650; p = 0.002). These findings indicated that the measurement of H-FABP might be suitable to predict the occurrence of myocardial injury and clinical events in patients with IGM.

There were several limitations in the current study. Firstly, this was an observational and prospective study, only association but not casual link could be determined. Secondly, we measured H-FABP once at baseline and have not compared with other traditional cardiac biomarkers. Thirdly, we did not assess all of the metabolic factors and parameters of insulin resistance due to the features of patients in our study. More studies may be needed to confirm our findings.

## Conclusions

Our study, for the first time to our knowledge, demonstrated that H-FABP was a useful predictor for adverse clinical outcomes in pre-diabetic patients with CAD, suggesting that elevation of H-FABP might provide prognostic information in CAD patients with mild abnormal glucose metabolism.

## Supplementary information

**Additional file 1: Table S1.** Baseline characteristics in study patients with and without events. **Table S2.** H-FABP levels and glucose metabolism status in relation to cardiovascular events.

## Data Availability

The datasets used and analyzed during the current study are available from the corresponding author on reasonable request.

## Abbreviations

H-FABP: Heart-type fatty acid-binding protein
CAD: Coronary artery disease
NGR: Normal glucose regulation
IGM: Impaired glucose metabolism
DM: Diabetes mellitus
CVE: Cardiovascular events
MetS: Metabolic syndrome
CAG: Coronary angiography
HbA1c: Hemoglobin A1c
MI: Myocardial infarction
CRV: Coronary revascularization
