# Supplementary material for: Prognostic utility of heart-type fatty acid-binding protein in patients with stable coronary artery disease and impaired glucose metabolism: a cohort study
Source: Cardiovasc Diabetol. 2020 Feb 10;19:15. doi: 10.1186/s12933-020-0992-0 (PMC7011523; doi:10.1186/s12933-020-0992-0)
Supplement: Supplementary file 1 — Additional file 1: Table S1. Baseline characteristics in study patients with and without events. Table S2. H-FABP levels and glucose metabolism status in relation to cardiovascular events. [file 12933_2020_992_MOESM1_ESM.docx]

**Table S1.** Baseline characteristics in study patients with and without events

| **Variables** | **Total** | **Events** | **Non-events** | **P value** |
| --- | --- | --- | --- | --- |
| **Baseline Characteristics** | | | | |
| Patients (n,%) | 4594 | 380(8.3%) | 4214(91.7%) |  |
| Age (years) | 58.2±9.9 | 60.1±10.1 | 58.0±9.7 | **<0.001** |
| Male (n,%) | 3266 (71.9%) | 263(69.2%) | 3003(71.3%) | 0.398 |
| BMI (kg/m^2^) | 25.92±3.14 | 26.02±3.25 | 25.92±3.13 | 0.533 |
| Hypertension (n,%) | 3005(65.4%) | 276(72.6%) | 2729(64.8%) | **0.002** |
| Dyslipidemia (n,%) | 4147(90.3%) | 335(88.2%) | 3812(90.5%) | 0.147 |
| NGR (n,%) | 801(17.4%) | 45(11.8%) | 756(17.9%) | **0.003** |
| Pre-DM (n,%) | 1904(41.4) | 125(32.9%) | 1779(39.8%) | **<0.001** |
| DM (n,%) | 1889(41.1%) | 210(55.3%) | 1679(39.8%) | **<0.001** |
| Current Smokers (n,%) | 1438(31.3%) | 95(25.0%) | 1343(31.9%) | **0.006** |
| Peripheral vascular disease (n,%) | 59(1.3%) | 8(2.1%) | 51(1.2%) | 0.138 |
| Cerebrovascular disease (n,%) | 145(3.2%) | 17(4.5%) | 128(3.0%) | 0.125 |
| Family history of CAD (n,%) | 643(14.0%) | 48(12.6%) | 595(14.1%) | 0.423 |
| **Laboratory data** | | | | |
| Triglyceride (mmol/L) | 1.80±1.23 | 1.86±1.32 | 1.80±1.23 | 0.585 |
| TC (mmol/L) | 4.14±1.17 | 4.18±1.11 | 4.14±1.17 | 0.518 |
| LDL-C (mmol/L) | 2.51±0.99 | 2.51±0.95 | 2.51±1.00 | 0.950 |
| HDL-C (mmol/L) | 1.05±0.29 | 1.07±0.26 | 1.05±0.29 | 0.379 |
| Glucose (mmol/L) | 6.20±2.04 | 6.57±2.13 | 6.17±2.03 | **<0.001** |
| HbA1c (%) | 6.54±1.24 | 6.89±1.39 | 6.51±1.22 | **<0.001** |
| H-FABP (ng/mL) | 2.45±1.86 | 2.95±2.52 | 2.41±1.79 | **<0.001** |
| Creatinine (umol/L) | 77.97±17.30 | 78.49±17.83 | 77.92±17.25 | 0.542 |
| eGFR (ml/min/1.73m^2^) | 93.74±9.12 | 92.27±9.40 | 93.87±9.09 | **0.001** |
| **Treatments in hospital** | | | | |
| Aspirin (n,%) | 4462(97.1%) | 365(96.1%) | 4097(97.2%) | 0.191 |
| β-Blokers (n,%) | 3417(78.3%) | 300(78.9%) | 3297(78.2%) | 0.748 |
| Lipid-lowering mediation (n,%) | 4269(92.9%) | 361(95.0%) | 3908(92.7%) | 0.100 |
| ACEI or ARB (n,%) | 2157(47.0%) | 213(56.1%) | 1944(46.1%) | 0.242 |
| **Gensini score** | 34.50±32.07 | 45.31±39.18 | 33.53±31.18 | **<0.001** |

CAD: coronary artery disease; BMI: Body mass index; NGR: normal glucose regulation; Pre-DM: pre-diabetes mellitus; DM: diabetes mellitus; TC: total cholesterol; LDL-C: low-density lipoprotein cholesterol; HDL-C: high-density lipoprotein cholesterol; HbA1c: glycated hemoglobin A1c; H-FABP: heart-fatty acid binding protein; ACEI: angiotensin converting enzyme inhibitors; ARB: angiotensin receptor blocker; eGFR: estimated glomerular filtration rate.

Values are expressed as the mean ± SD or n (%). Bold values indicate statistical significance.

**Table S2.** H-FABP levels and glucose metabolism status in relation to cardiovascular events

| **Variable** | **HRs(95%CIs)** | | | | |
| --- | --- | --- | --- | --- | --- |
|  | **Unadjusted** | **P value** | | **Adjusted******* | **P value** |
| **Glucose metabolism status** |  |  | |  |  |
| **NGR** | Reference | - | | Reference | **-** |
| **Pre-DM** | 1.128(0.802-1.586) | 0.488 | | 1.016(0.649-1.435) | 0.885 |
| **DM** | 2.011(1.457-2.775) | **<0.001** | | 1.608(1.151-2.248) | **0.005** |
| **H-FABP levels** |  |  | |  |  |
| **Tertile 1** | Reference | - | Reference | | - |
| **Tertile 2** | 1.220(0.935-1.592) | 0.143 | 1.136(0.864-1.493) | | 0.361 |
| **Tertile 3** | 1.670(1.299-2.146) | **<0.001** | 1.335(1.011-1.762) | | **0.041** |

NGR: normal glucose regulation; Pre-DM: pre-diabetes mellitus; DM: diabetes mellitus; H-FABP: Heart-type fatty acid-binding protein; HRs: hazard ratios; CIs: confidential intervals. Bold values indicate statistical significance. *Adjusted for age, gender, hypertension, dyslipidemia, body mass index, current smoking, family history of CAD, Gensini score and eGFR.
